# Supplementary material for: Inertial displacement of a domain wall excited by ultra-short circularly polarized laser pulses
Source: Nat Commun. 2017 May 17;8:15226. doi: 10.1038/ncomms15226 (PMC5442316; doi:10.1038/ncomms15226)
Supplement: Supplementary Information — Supplementary Figures, Supplementary Notes and Supplementary References. [file ncomms15226-s1.pdf]

Supplementary Information:
Inertial displacement of a domain wall excited by ultra-short circularly polarized laser pulses

February 24, 2017

Supplementary Note 1:
Elastic bubble expansion and geometrical pinning

To describe the geometrical DW pinning exploited in our experiment we employ a simple DW propagation model where the motion of a DW of negligible width is determined by the competition between DW energy $E_\sigma = \sigma \cdot t \cdot l$ and Zeeman energy $E_\mathrm{Z} = -2M_\mathrm{S} \cdot H_\mathrm{A} \cdot t \cdot S$. $\sigma = 4\sqrt{AK_\perp}$ is the DW energy per unit area, M_S, A, and $K_\perp$ are saturation magnetisation, exchange stiffness and effective out-of-plane uniaxial anisotropy constants, respectively, l is the DW length, S is the area of the reversed domain and t is the thickness of the magnetic layer. The 'friction' of DW motion generated by DW pinning on defects in the magnetic film is described in our model by a coercive *intrinsic* propagation field H_P which is considered to be everywhere the same. We also neglect the effect of magnetic pseudo-charges generated by DW deformation, since the radius of curvature is much larger than the width of the DW in our micrometer wide Hall-crossbar structures. We therefore consider magnetic field driven DW propagation to be governed only by the competition between DW energy and Zeeman energy.

We first consider a circular-shaped domain of radius r expanding around a nucleation center

in a magnetic plate without any geometrical restrictions. Minimizing the total energy $E_{\mathrm{TOT}} = E_\sigma + E_{\mathrm{Z}}$ yields a DW with minimal length bordering a maximal area of the reversed domain. If the wall propagates by dq, the total energy changes by

$$\frac{dE_{\mathrm{TOT}}}{dq} = \sigma t \frac{dl}{dq} - 2M_{\mathrm{S}}H_{\mathrm{A}}t\frac{dS}{dq} \equiv 2M_{\mathrm{S}}(H_{\mathrm{R}} - H_{\mathrm{A}})2\pi r t, \tag{1}$$

where $H_{\mathrm{R}} = (2M_{\mathrm{S}})^{-1}\sigma/r$ is hereafter defined as the *virtual* restoring field. The effective net field H_{NET} oriented along the layer normal direction, which induced the DW propagation, contains in addition to the applied field H_{A}, the propagation field H_{P} and the damping torque field H_α (*1*) as well as the contribution H_{R} arising from the DW curvature (*2*).

In our geometrically constricted Hall-crossbar of width w, we can also reduce the DW motion to a one dimensional problem, that of a virtually straight DW propagating at the position q with the velocity v of the real DW center, and on which a restoring force acts due to the curvature of the real wall. Within the bar outside of the Hall cross, the DW of $l = w$ stays straight perpendicular to the stripe boundaries, and propagates in the applied magnetic field. When the DW reaches the two corners of the cross entrance, the DW must increase its length to continue propagation. The energetically optimal way is a bubble like expansion as shown in Supplementary Fig. 1 where the DW starts as a flat line (stage 'B') and remains connected to the corners of the cross entrance until it coincides with a semi-circle of radius $w/2 \equiv d$, (stage 'C'). During this process where the DW propagates from the position $q = -d \to 0$, the DW length enhances as $l = 2r \cdot \arcsin(d/r)$ and the reversed domain surface increases as $S = r^2\left[\arcsin(d/r) - (d/r)\sqrt{1-(d/r)^2}\right]$ since the DW curvature radius shrinks from $r = \infty \to d$. The relation between r and q is given by $q = r - d - \sqrt{r^2 - d^2}$. The total energy variation is given by

$$\frac{dE_{\mathrm{TOT}}}{dq} = (\sigma - 2M_{\mathrm{S}}H_{\mathrm{A}}r)t\frac{\sqrt{r^2-d^2}\arcsin(d/r) - d}{\sqrt{r^2-d^2} - r}. \tag{2}$$

Beyond the semi-circle (stage 'C'-'D'), the two entrance corners do no longer influence the DW propagation and the domain continues to expand circularly with increasing radius. The relation between curvature radius r and propagation coordinate q is now $r = q + d$ and the energy variation leads to

$$\frac{dE_{\mathrm{TOT}}}{dq} = (\sigma - 2M_{\mathrm{S}}H_{\mathrm{A}}r)t\pi. \tag{3}$$

The equilibrium condition $dE_{\mathrm{TOT}}/dq = 0$ leads to the applied magnetic field at which restoring and driving forces are balanced: $H_{\mathrm{R}}(q) = \frac{\sigma}{2M_{\mathrm{S}} \cdot r'}$ within the range of $q = -w/2 \rightarrow 0$, $q = r' - d - \sqrt{r'^2 - d^2}$ and $H_{\mathrm{R}}(q) = \frac{\sigma}{2M_{\mathrm{S}}[q+d]}$ within the range of $q = 0 \rightarrow (\sqrt{5} - 1)d$, hence, the maximum of the restoring field

$$H_{\mathrm{R}}^{\max} = \frac{\sigma}{M_{\mathrm{S}}w} \tag{4}$$

is reached, when the DW reaches the cross center at $q = 0$.

According to our model, both virtual restoring field H_{R} and the intrinsic propagation field H_{P} oppose the DW propagation. Thus, DW propagation without thermal activation will start as soon as the applied field exceeds the value of $H_{\mathrm{R}} + H_{\mathrm{P}}$. In contrast to H_{P}, the restoring field $H_{\mathrm{R}}(q)$ is a function of DW position and the model predicts a maximum restoring field at the center of the cross. Since $H_{\mathrm{R}}^{\max}$ is inverse proportional to the bar width, larger pinning in narrower bars is expected. Indeed, our experimental findings shown in the main text (Fig. 2) are in very good agreement with the theoretical pretictions of our model and confirm that the geometrical pinning dominates by far the intrinsic pinning and also possible pinning at the pair of exit corners (stage 'D') due to demagnetisation field inhomogeneities (*2*).

In the same spirit, virtual restoring field and intrinsic pinning on defects oppose DW motion when the driving mechanism is optical spin-transfer torque generated by circularly polarised laser pulses (LPs). This allows us to relate a measure of the oSTT driven domain wall motion to the geometrical pinning strength which is always measured as a reference quantity in a field

assisted depinning experiment without irradiation. In Supplementary Fig. 2, we present data of polarisation dependent depinning fields B_{dp} of 2, 4 and 6 μm wide crossbar devices as a function of LP energy density. Without laser irradiation, B_{dp} is largest for the narrowest bar. Thermal heating by the LP irradiation, however, is more efficient for the narrower device so that the reduction of B_{dp} is faster with increasing LP energy density for the narrower bars. We performed experiments also at a higher and lower temperature, Supplementary Fig. 2 C,D. The comparison of the 75K and 95K measurements, shown below, indeed confirms that oSTT is present independently of the sample temperature. At lower temperatures, depinning without additional applied magnetic field is realised at even lower laser powers.

Supplementary Note 2: Micromagnetic method

2.1: Landau-Lifshitz-Bloch approach (LLB)

We state again here firstly the governing equations as the forthcoming description is centered around them: The time evolution of the magnetization **m** in the LLB approach (*3*) and the spin-density **s** (*4*) read :

$$\frac{\partial \mathbf{m}}{\partial t} = -\gamma \mathbf{m} \times \mathbf{H}_{\text{eff}} + \mathbf{\Gamma}_{\text{tr}} + \mathbf{\Gamma}_{\text{lt}} \tag{5}$$

$$\frac{\partial \mathbf{s}}{\partial t} = \frac{-J_{\text{ex}}}{\hbar m_{eq}} \mathbf{s} \times \mathbf{m} + R(t)\hat{\mathbf{n}} - \frac{\mathbf{s}}{\tau_{\text{rec}}} \tag{6}$$

We start by describing Eq.(5): There, **m** is the magnetization at temperature T, normalized by the zero temperature saturation magnetization M_0 , γ is the gyromagnetic ratio and $\mathbf{H}_{\text{eff}}$ is the effective field (as described later on). M_0 was determined by extrapolation of SQUID-data and is here 35.5 kA/m. The first term on the right hand-side of Eq. (1) describes the precession of **m** around $\mathbf{H}_{\text{eff}}$ while the second term $\mathbf{\Gamma}_{\text{tr}}$=$-\frac{\gamma\alpha_\perp}{m^2}\mathbf{m} \times (\mathbf{m} \times \mathbf{H}_{\text{eff}})$ is the transverse torque with an associated damping $\alpha_\perp(T)$=$\lambda\left(1 - \frac{T}{3T_{\text{C}}}\right)$, resulting in relaxation of **m** into the direction of $\mathbf{H}_{\text{eff}}$. Here, λ is the microscopic damping parameter at T=0.

The first and second terms on the right hand-side of Eq.(5) constitute the torques included in the LLG description. In the LLB equation, a third term, $\mathbf{\Gamma}_{\text{lt}}=\frac{\gamma\alpha_{||}}{m^2}\left(\mathbf{m}\cdot\mathbf{H}_{\text{eff}}\right)\mathbf{m}$ is present, allowing for a longitudinal variation of **m**; in other words $|\mathbf{m}|$ is not conserved and is allowed to fluctuate with an associated damping parameter $\alpha_{||}(T)=\frac{2T\lambda}{3T_{\text{C}}}$ beacuse, at elevated temperatures, all atomic spins whose ensemble form the corresponding **m** in a computational cell, are not necessarily all parallel to each other at all times (which is the assumption and a constraint in LLG-micromagnetics). Further, the interaction terms taken into account here result in $\mathbf{H}_{\text{eff}}=\mathbf{H}_{\text{d}}+\mathbf{H}_{\text{ex}}+\mathbf{H}_{\text{mf}}+\mathbf{H}_{\text{k}}+\mathbf{H}_{\text{OSTT}}+\mathbf{H}_{\text{r}}$, which are, demagnetizing, exchange, internal material field, uniaxial magnetocrystalline anisotropy, optical spin transfer torque and geometrical pinning -fields ($\mathbf{H}_{\text{r}}(x)$ is taken directly from Fig. 3 A in the main text), respectively. The effective field terms are evaluated from the free energy density functional f as $\frac{-1}{\mu_0 M_0}\frac{\delta f}{\delta \mathbf{m}}$. Its temperature dependence is described in terms of the thermodynamic equilibrium functions of the pertinent material parameters; normalized equilibrium magnetization m_{eq} at a given T (normalized by M_0), exchange stiffness $A(T)$, uniaxial magnetocrystalline anisotropy, $K_{\perp}(T)$, $K_{||}(T)$ and longitudinal susceptibility $\chi_{||}(T)$. Here, the temperature dependence of m_{eq} was evaluated within the mean field approximation, by a Langevin function fit to measured SQUID-data and $\chi_{||}(T)$ was calculated as shown below. The equilibrium magnetization at $T = 90$ K is here 18 kAm^{-1}. $K_{||}$ at the temperature used in the simulations was estimated from data presented in De Ranieri et al. (*5*) taken on nominally identical GaMnAsP material. The mean values of $K_{||}$=350 Jm^{-3} and $K_{\perp}$=1.51 kJm^{-3} were determined from characterization measurements on single bar-devices at a temperature of $T = 90$ K, and the exchange stiffness constant of $A(T = 90\text{ K}) = 50$ fJm^{-1} is reasonable for $Ga_{0.94}Mn_{0.06}As_{0.9}P_{0.1}$. Implementing the values above in our bubble like DW propagation model reproduces the measured depinning fields at various bar widths. By using previously measured values valid for this temperature we avoid mean-field fitting for most material parameters and thus we are more certain of their realistic values. For dynamical simu-

lations we choose the damping parameter λ=0.01. All simulations are performed considering a base temperature of $T = 90$ K (in accordance to most of the experimental measurements). The demagnetizing field is divided into near-field and the far-field contributions and is described in terms of the demagnetizing tensor, $\hat{N}$ in the standard manner; the dipole field at point $\mathbf{r}_\mathrm{i}$ from all dipoles at points $\mathbf{r}_\mathrm{j}$ is $\mathbf{H}^\mathrm{i}_\mathrm{d}(T)$=$-m_\mathrm{eq}M_0\sum_\mathrm{j}\hat{N}(\mathbf{r}_\mathrm{i}-\mathbf{r}_\mathrm{j},\Delta_\mathrm{x},\Delta_\mathrm{y},\Delta_\mathrm{z})\mathbf{m}_\mathrm{j}$, where $\Delta_\mathrm{x,y,z}$ are the dimensions of the discretization cells used along x, y and z, respectively. For the near-field, $\hat{N}$ is evaluated by the analytical formulae for interactions between tetragonal cells as derived by Newell, Williams and Dunlop (*6*). For the far-field (here, for inter-cell distances $\geq$ 40 cells), the kernel elements of $\hat{N}$ correspond to those for point dipoles. $\hat{N}$ need only to be computed once and stored in memory. The form of $\mathbf{H}^\mathrm{i}_\mathrm{d}(T)$ is that of a spatial convolution. This convolution is then evaluated by standard FFT-techniques. The exchange field $\mathbf{H}_\mathrm{ex}(T)$=$\frac{2A(T)}{\mu_0 m^2_\mathrm{eq}M_0}\partial^2_\mathbf{r}\mathbf{m}$, where the second derivative is computed by a finite difference three-point stencil in each spatial direction. $\mathbf{H}_\mathrm{mf}$, responsible for stabilizing $|\mathbf{m}|$ is determined by the parallel susceptibility $\chi_{||}(T)$ as $\mathbf{H}_\mathrm{mf}(T)$=$\frac{1}{2\chi_{||}(T)}\left(1-\frac{m^2}{m^2_\mathrm{eq}}\right)\mathbf{m}$ with $\chi_{||}=(\partial m_\mathrm{eq}/\partial H)_{H\to 0}$ and H being the magnitude of an applied field. In this work, the global easy axis $\hat{\mathbf{u}}||\hat{\mathbf{z}}$ and the in-plane uniaxial anisotropy axis $\hat{\mathbf{u}}||\hat{\mathbf{y}}$. Each anisotropy term contributes to $\mathbf{H}_\mathrm{k}(T)$ as $\mathbf{H}_\mathrm{k}(T)$=$\frac{2K(T)}{\mu_0 m^2_\mathrm{eq}(T)M_0}(\mathbf{m}\cdot\hat{\mathbf{u}})\hat{\mathbf{u}}$. The last effective field term, $\mathbf{H}_\mathrm{r}(T)$ is based on the considered pinning field profile for a bubble domain pinned at a cross, while the effect of temperature is taken into account by considering the reduction of $|\mathbf{B}_\mathrm{dp}|$ for σ^0-light at the laser fluency corresponding to DW depinning for σ^+-light. Therefore, the maximum $|\mathbf{H}_\mathrm{r}(T)|$ used in the simulation corresponds to 0.1 mT. As it acts as to pull the DW in the opposite direction of its excited motion, then in the simulations, the direction of the virtual restoring field $\mathbf{H}_\mathrm{r}(x)$ is along the $\mp z$-direction if the DW moves along the $\pm x$-direction. Finally, the boundary condition used for $\mathbf{m}$ on all free surfaces is $\frac{\partial\mathbf{m}}{\partial\hat{\mathbf{r}}_\mathrm{n}}=0$, where $\hat{\mathbf{r}}_\mathrm{n}$ is the outward unit normal.

We now turn to Eq.(6) and its coupling to Eq.(5). Here, J_ex is the exchange coupling between

photo-induced electrons and the local magnetization $\mathbf{m}$. We use J_{ex}=$JS_{\text{Mn}}c_{\text{Mn}}$, where J=10 meVnm^3, S_{Mn}=5/2 is the local Mn-moment and $c_{\text{Mn}} \sim 1$ nm^{-3} is the typical moment density (*?*). When coupling to the LLB-equation we assume a temperature variation of the effective exchange coupling to the macro-vector **m** at increased T and for simplicity assume $J_{\text{ex}} \rightarrow J_{\text{ex}}m_{\text{eq}}^2$. The first term on the right hand-side of Eq.(6) describes the precession of **s** around the exchange field produced by **m** (in this step the effect of **m** on **s** is established) while the second term gives the injection of spin-polarized electrons with R being the rate per unit volume and $\hat{n}$ the initial spin polarization direction defined by the helicity of the light with $\hat{\mathbf{n}}$=[00$\pm$1]. Finally, the third term represents the decay of the photo-carrier spin with a life-time of τ_{rec}, limited in our case by the carrier-recombination time. Based on previous measurements in literature, we set τ_{rec}=30 ps. Gradient terms in **s** are neglected. During precession, **s** transfer its angular momentum to **m**. The precession time of **s** is very fast as compared to the natural precession of **m** ($\sim$ 100 fs versus $\sim$ 1 ns). The absorption of angular momentum from **s** results in a torque on **m**. This torque is then entered into Eq.(5) by an augmentation to the rest of the effective field by $\mathbf{H}_{\text{OSTT}}$ (thus the effect of **s** on **m** is established); The interaction energy density between **s** and **m** is f_{ex}=$\frac{-J_{\text{eff}}(T)}{m_{\text{eq}}}\mathbf{s}\cdot\mathbf{m}$. The corresponding effective field term is then according to the definition in the preceeding paragraph, $\mathbf{H}_{\text{OSTT}}(T)$=$\frac{J_{\text{eff}}(T)}{\mu_0 m_{\text{eq}} M_0}\mathbf{s}$. Equations (1) and (2) are solved together using a 5th order Runge-Kutta integration scheme.

2.2: Computational geometry and simulation procedure

We consider a one-dimensional bar with 4095 x 1 x 1 computational cells composing a structure as shown in Supplementary Fig. 3. The cell dimension is 4 nm x 4 μm x 25 nm. A Bloch DW is initialized in the center of the bar and let to relax quickly with strong damping by setting λ=0.9. This configuration is then used as a starting configuration for the simulations of domain wall motion under the light pulses.

Once the domain wall is prepared, circularly polarized light is pulsed at a rate of 80 MHz. The length of each pulse is set to 150 fs. For the simulation of the depinning process, the spin-polarized carrier injection rate is $R = 1.225 \times 10^{39}\mathrm{m}^{-3}\mathrm{s}^{-1}$. This order of magnitude for R is required for the DW to escape the elastic pinning potential. The equivalent pulse power corresponds to the time-averaged laser power used in the experiments of the order of 20 mW assuming a skin depth of $1\ \mu$m. Further, all simulations were done in zero externally applied magnetic field and a damping of $\lambda = 0.01$ was used in all dynamical simulations.

Throughout all simulations a centering procedure is employed, that keeps the DW in the middle of the length of the bar. In this way, propagation distances as long as needed can be simulated without having to worry about stray field effects should the domain wall have come close to the edges of the bar or that the DW moves out of the computational region.

Supplementary Fig. 4 shows the calculated time-averaged DW velocity v_{DW} as a function of LP energy density. We identify the Walker breakdown peak velocity of about 5 m/s at about 18 mJ/cm^2 for the DW with only out-of-plane magnetic anisotropy. Stabilising the DW structure, e.g., by DMI or by introducing additional anisotropy via mechanical strain, can shift the WB to higher values and allow for achieving higher DW velocities, (*5*). We show in Supplementary Fig. 4 that indeed higher velocities can be achieved when the static structure of the DW is stabilised by introducing additional uniaxial in-plane anisotropy. We finally note that the limiting speed for any texture to propagate in a given magnetic material is the magnon group velocity $\mathrm{v_m}$. We estimate $v_{\mathrm{m}} = 4 \cdot J \cdot S_{\mathrm{Mn}} \cdot a/\hbar$ using the parameter of our GaMnAsP film at $T = 90$ K, a is the spin separation length and J is the next neighbour exchange constant. With $\mathrm{J_{ex}} = A \cdot a/(2S_{\mathrm{Mn}}^2)$ and $a = (2 \cdot g \cdot \mu_{\mathrm{B}} \cdot S_{\mathrm{Mn}}/M_S)^{1/3}$ (g is the Lande factor and μ_{B} is the Bohr magneton) we obtain $v_{\mathrm{m}} \approx 1\ \mathrm{km/s}$.

Supplementary Note 3: Additional experimental evidences for optical spin transfer torque driven DW motion

3.1: Wavelength dependency of helicity dependent DW motion

We have performed wavelength dependent experiments to support the optical spin transfer torque origin of the helicity dependent LP induced DW motion. In experiments described in the main text we use LP excitation with a wavelength λ = 750 nm that excites photo-electrons slightly above the bottom of the GaAs conduction band so that for a circularly polarized incident light, photo-electrons become spin-polarized with the degree of polarization approaching the maximum theoretical value of 50% (*7*). At energies above and below band gap energy (Supplementary Fig. 5), the spin polarisation of the photo-electrons is reduced. Photons excitation at higher energies results in the reduction of net-photoelectron spin-polarisation mainly because carrier excitation from the split-off valence band can take place. The photocarrier generation from low-energy photons with sub-band gap energies comes mainly from excitation of impurity states within the band gap.

3.2: Dependency of helicity dependent DW motion on the sweep direction of the focused laser spot

We now investigate the effect of the thermal gradients generated by the laser spot on the helicity dependent DW depinning. By inverting the sweep direction of the focused LP spot with respect to the geometrically pinned DW, we invert also the thermal gradient affecting the DW. In case that the LP spot approaches from the reversed domain along the patterned bar, Supplementary Fig. 6A, both $B_{\text{dp}}(\sigma^+)$ and $B_{\text{dp}}(\sigma^-)$ decrease faster compared to the situation where the laser spot approaches from the unreversed domain (Supplementary Fig. 6B). This observation is explained by the stronger temperature increase from LP heating in the narrow bar compared to

the lower temperature rise in the wider cross area. On the other hand, the helicity dependence of B_{dp}, which is of oSTT origin, remains unaffected by the LP sweep direction.

3.3: Continuous wave excitation vs. laser pulse excitation

We now show that the DW can be also moved by a focused laser spot of continuous wave (cw) excitation. To compare the efficiency of the cw-excitation with our ultrashort LP-excitation approach we have deduced $B_{\mathrm{dp}}(\sigma^{+})$ and $B_{\mathrm{dp}}(\sigma^{-})$ for LP- and cw-excitation at the same averaged laser power P_{av}. Based on our LLB approach, we also have calculated DW propagation driven by oSTT from LP- and cw- excitations. From the simulations, we have derived the averaged DW velocity vs. P_{av} at the position of maximal restoring field (Supplementary Fig. 7A). Positive averaged velocities correspond therefore to the depinning of the DW. For zero or negative velocities, the DW remains pinned. Both calculation and experimental results presented in Supplementary Fig. 7 confirm that a DW can be depinned via oSTT generated by ultrashort LP and by cw-excitation. However, cw-driven DW propagation requires always higher averaged laser power (Supplementary Fig. 7A). Comparing $B_{\mathrm{dp}}(\sigma^{-}, \sigma^{+})$ (Supplementary Fig. 7B, C) for the two excitation schemes at equal averaged laser power shows a stronger efficiency of the pulsed oSTT. Moreover, the effect of laser heating on DW depinning is stronger in case of cw-excitation compared to ultrashort LP-excitation. Therefore, helicity dependent inertial DW motion induced by ultrashort LP is more efficient than DW motion induced by constant excitation.

3.4: Temperature dependent depinning field

The temperature dependence of the resistance of the magnetic bar is used to monitor and control the actual sample temperature (Supplementary Fig. 8A). In order to obtain the accurate resistivity dependence of our devices we performed a reference measurement in a bath cryostat, where

the (Ga,Mn)(As,P) sample is thermally anchored to a calibrated temperature sensor and where the temperature dependent resistivity $R(T)$ of (Ga,Mn)(As,P) is monitored during heating-up from 4 K to room temperature. The Curie temperature $Tc = 115$ K is obtained by identifying the cusp in dR/dT (*8*), (Supplementary Fig. 8A). We have determined B_{dp} without laser irradiation at a 4 μm wide device as a function of temperature in a temperature range below $T = 90$ K until close to Curie-temperture (Supplementary Fig. 8B). This allows us to estimate an effective sample temperature deduced from the comparison between temperature dependent measurements of B_{dp} without irradiation and measurements of $B_{\text{dp}}(\sigma^0)$ vs. P_{av} laser power and at fixed base temperature (*9*).

3.5: MCD induced temperature gradients

In this section, we identify the sign and estimate the magnitude of the temperature gradient generated by MCD between two opposite magnetized domains. We use a 6 μm wide, 18 μm long (contact-to-contact) bar-device patterned from our GaMnAsP/GaAs film. LPs are focused at the center of our bar as shown in Supplementary Fig. 9. We evaluate the temperature variation at opposite saturation magnetisations from the resistance variation detected in our sample at fixed LP polarisation. We employ a sensitive double Lock-in technique and compare magnetisation dependent resistance variation with the temperature dependence of the GaMnAsP film resistivity.

To estimate the temperature variation at the irradiated spot position from the resistance variation measured in 4-point geometry over the whole bar device, we employ a simple resistor network model described in Supplementary Fig. 10. The 18 μm long and 6 μm wide bar is divided into 27 (2 μm $\times$ 2 μm) squares. Only the central square is irradiated having the resistance R^L_{sq} . All other squares remain in darkness having the equal resistances, R^D_{sq}. Within this simple resistor network model, we can estimate the temperature variations

generated by the MCD at the irradiated square of the device by detecting the total device resistance variation at opposite saturation magnetisations when comparing the measured data to a reference measurement of the temperature dependency of $\Delta = R(T)/R(90\text{ K})$, Supplementary Fig. 10, right. Since only the irradiated spot can be compared with this reference measurement, we need to relate the sample temperature variation of the spot to the measurable resistance variation of the total device as shown in Supplementary Fig. 10 (left). In the frame of this approximation, the ratio of irradiated and non-irradiated square resistances is estimated to $\Delta = R^L_{sq}/R^D_{sq} = 27R^L_T/R^D_T - 26$; ($R^L_T$, and R^D_T are the measurable total bar device resistances with and without spot irradiation). To avoid any small alternations of our focused LP spot in intensity, spot position, etc., we measure the resistance variation due to the MCD by changing periodically the sample saturation magnetisation (at a frequency of 0.2 Hz) and keeping simultaneously the LP polarisation fixed. A first Lock-in amplifier measures the 4-point resistance as the response to an alternating probe current (f = 123 Hz). After the subtraction of an offset, the output of the first Lock-in is amplified by a factor x100. The resulting signal feeds into a second Lock-in, which amplifies the signal with reference to the alternating saturation magnetisation. As a result, we obtain $\Delta R_m = R^L_{T,+M_z} - R^L_{T,-M_z}$. We evaluate $dT = T(R^L_{sq,+M_z}) - T(R^L_{sq,-M_z}) = \alpha(\Delta_{+M_z} - \Delta_{-M_z})$, with $T = \alpha\Delta$ and α is obtained from the linear slope of the temperature dependence of the sample resistance around $T = 90$ K. Since $\Delta_{+M_z} - \Delta_{-M_z} = 27(R^L_{T,+M_z} - R^L_{T,-M_z})/R^D_T = 27\Delta R_m/R^D_T$, we can estimate $dT = 27\alpha\Delta R_m/R^D_T$ with $R^D_T = 1.244\text{ k}\Omega$.

In Supplementary Fig. 11(a,b) we show the LP polarisation dependent temperature variation at the irradiated spot between positive and negative saturation magnetisations. At $T = 90\ K$ substrate temperature, the temperature variation is of the order of $+(-)200\ mK$ for circularly polarised $\sigma^+(\sigma^-)$ LPs. Hence, the $\sigma^+(\sigma^-)$ LPs irradiated film with positive (negative) magnetisation becomes hotter than the film with negative (positive) magnetisation orientation.

In our oSTT experiments shown in Fig. 3 of the main text, we saturate the magnetic bar in a strong negative magnetic field and nucleate a reversed domain at the left side of the bar with positive magnetisation orientation . When irradiated with $\sigma^+(\sigma^-)$ polarised LPs, MCD heats up the nucleated magnetic domain more (less) than the rest of the magnetic film with negative magnetization orientation. In the experiment, we observe DW depinning and motion towards the area with negative magnetisation only when the DW is irradiated with σ^+ polarised LPs. If we repeat the experiment with inverted magnetisation (positive saturation, nucleated domain with negative magnetisation orientation) the DW moves towards the positive magnetisation orientation only when irradiated with σ^- polarised LPs. Hence, we always observe that the circularly polarised LP exposed DW moves towards the colder region, which excludes the MCD origin of the observed DW motion. Considering a DW width of ~ 50 nm (Supplementary Fig. 11C), we estimate a MCD generated heat gradient of $\sim 6 \times 10^6$ K/m. This value is smaller than the heat-gradient generated by the focused light spot independent of polarisation.

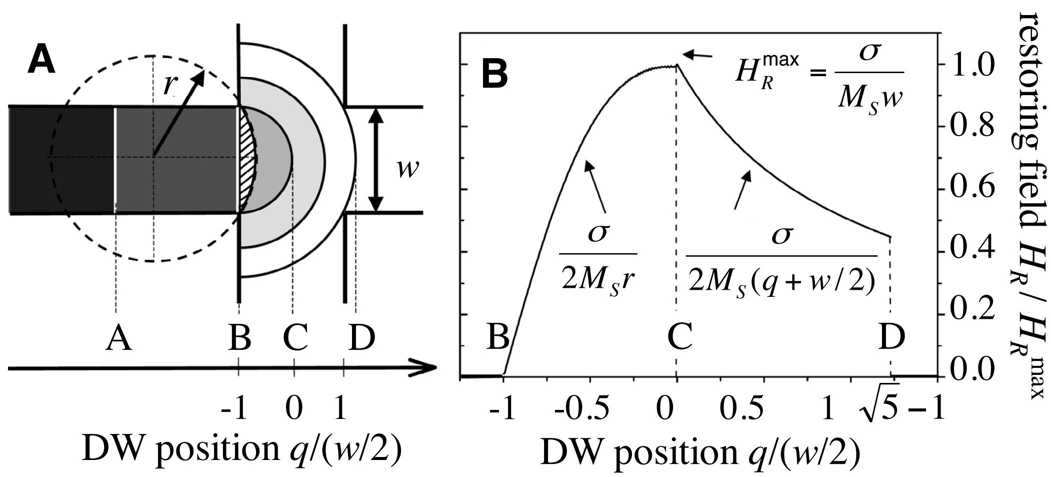

Supplementary Fig. 1: Geometrical domain wall pinning.

(A) "Soap-bubble" like expansion of a DW within a symmetric cross. The DW stays pinned on the cross input corners until stage 'C' is reached. At this position, the geometrical restoring field H_R reaches its maximum. (B) Position dependent *virtual* restoring field $H_R(q)$ arising from the wall curvature is reflecting the elasticity of the wall. Introducing $H_R(q)$ reduces our system to a one-dimensional problem, that of a virtual straight DW propagating with position q and velocity v of the real DW center.

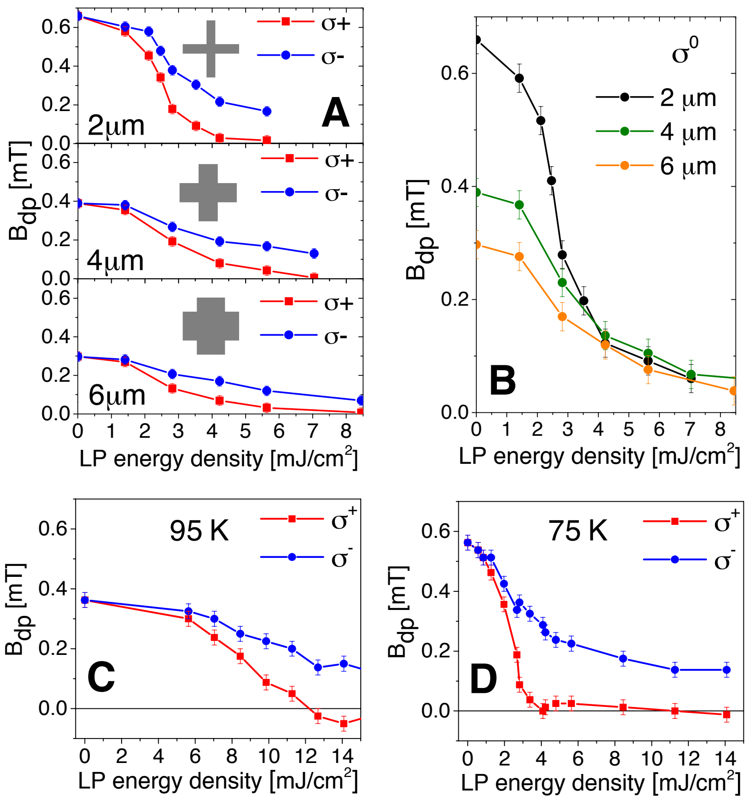

Supplementary Fig. 2: LP dependent depinning field.

Polarization dependent depinning field $B_{\text{dp}}(\sigma^+, \sigma^-, \sigma^0)$ as a function of LP energy density of 2, 4 and 6 μm wide crossbars for (A) circularly ($\sigma^{+,-}$) and (B) linearly (σ^0) polarized LPs. The difference between $B_{\text{dp}}(\sigma^+)$ and $B_{\text{dp}}(\sigma^-)$ is due to the optical spin transfer torque and the decrease of B_{dp} with increaseing LP energy density is due to the heating from photon absorption. Circularly polarization dependent depinning field $B_{\text{dp}}(\sigma^+, \sigma^-)$ measured at the 4 μm wide crossbars device at different sample temperatures, $T = 95K$ (C) and at $T = 75K$ (D) The error bars correspond to the maximal observed scatter of B_{dp} around the corresponding mean values.

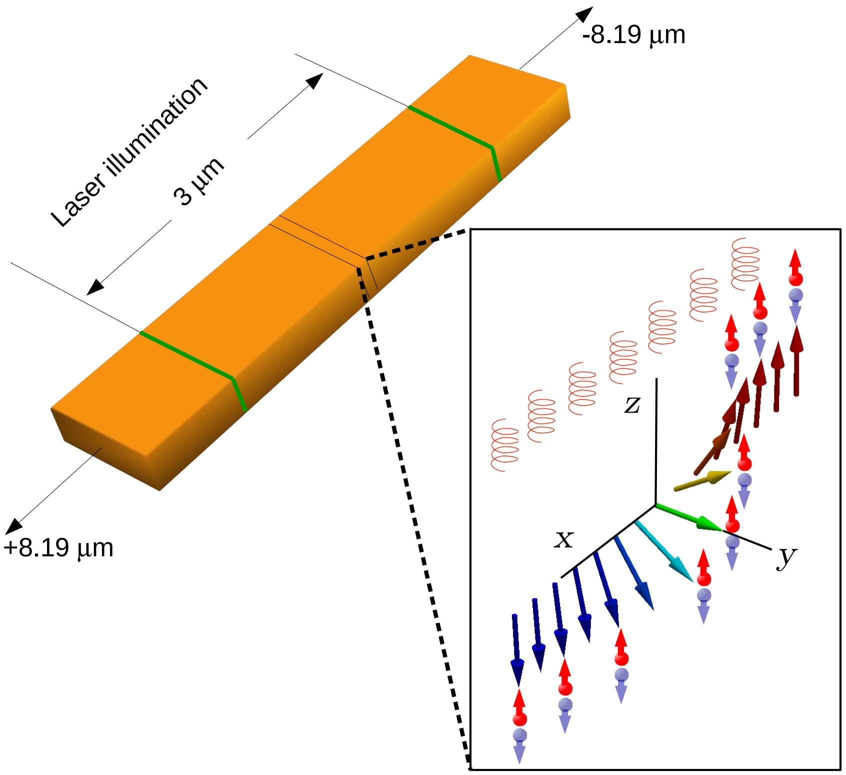

Supplementary Fig. 3: Computational Setup.

The total bar extension is 4x4095=16384 nm along x, 4 μm wide and 25 nm thick. The DW is of Bloch type, initially located at the center of the bar. Circularly polarized light is applied at constant fluency within a 3 μm long window around the domain wall. The blow-up shows the computed structure of the DW and schematically shows the spin up or spin down spin-polarized charge carriers generated by the circularly polarized light.

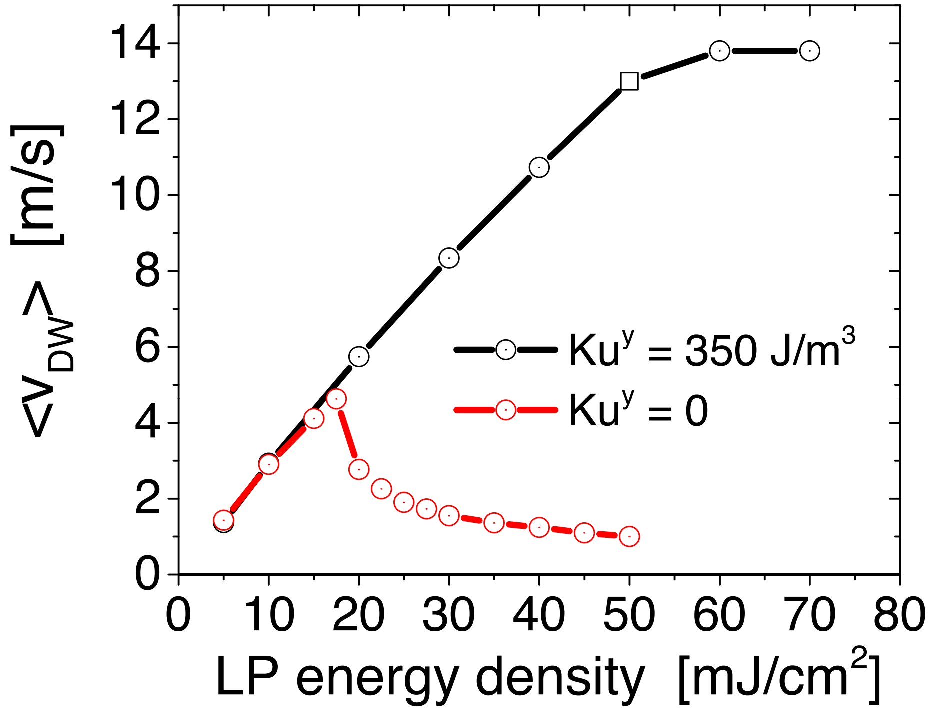

Supplementary Fig. 4: Domain Wall mobility.

DW velocity as a function of LP energy density calculated in the LLB approach. DW velocity, time averaged during the 12.5ns dark phase after a oSTT pulse, as a function of laser power for GaMnAsP film without (red) and with uniaxial inplane anisotropy Kuy (black). The uniaxial inplane anisotropy can be introduced by mechanical strain, (*5*).

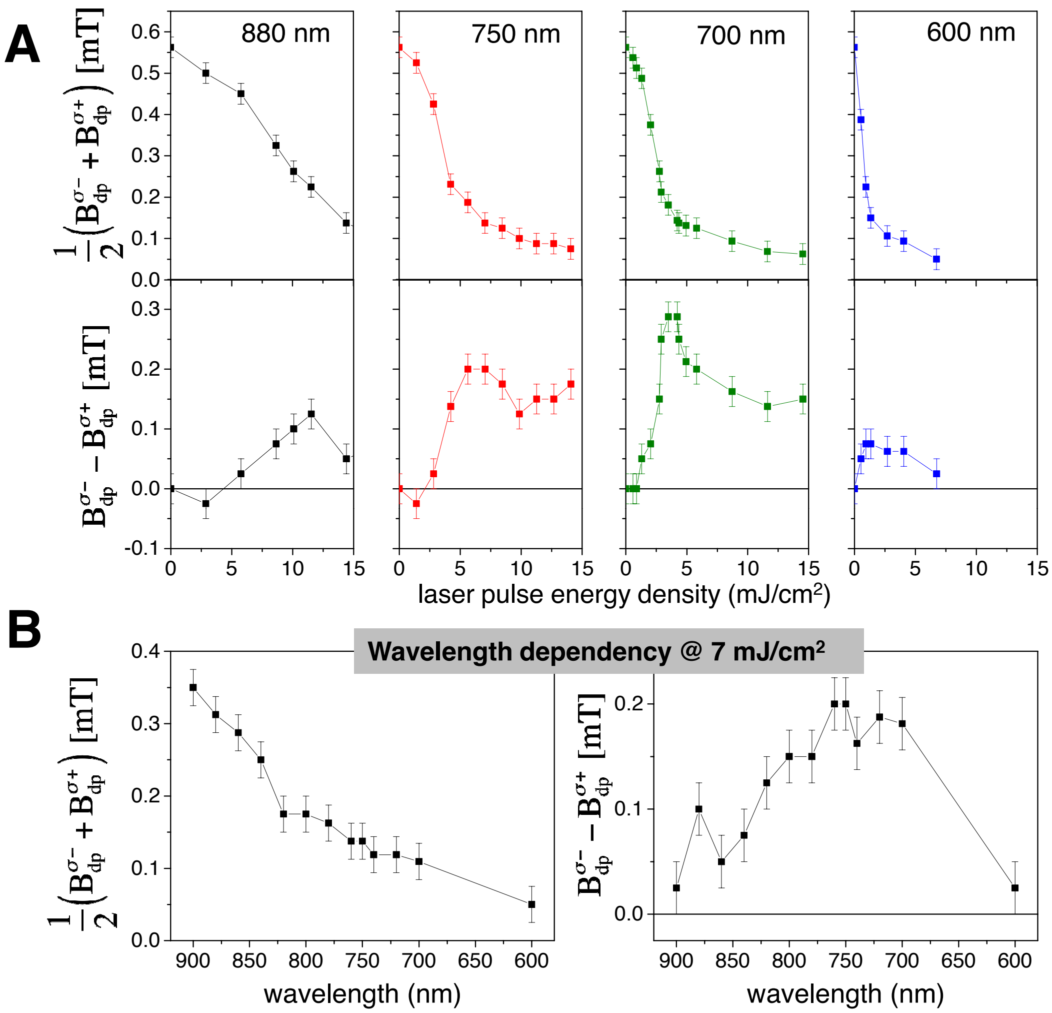

Supplementary Fig. 5: Wavelength dependent domain wall depinning.

(A) Average and difference of the depinning field $B_{\rm dp}$ for σ^- and σ^+ vs. LP energy density at various wavelengths at 75 K sample temperature. The average $1/2[B_{\rm dp}(\sigma^-)+B_{\rm dp}(\sigma^+)]$ indicates the reduction of the geometrical pinning due to helicity independent LP heating. The difference $B_{\rm dp}(\sigma^-) - B_{\rm dp}(\sigma^+)$ shows the effect of the oSTT on the DW motion. (B) The average at a fixed LP energy density of 7 mJ/cm^2 identifies the reduction of geometrical DW pinning with increasing LP heating due to enhanced absorption at higher photon energy. On the other hand, the difference $B_{\rm dp}(\sigma^-) - B_{\rm dp}(\sigma^+)$ shows that the oSTT efficiency is highest when the photon energy is close to the band gap of GaAs and it is strongly suppressed when photo-electrons are generated from the spin-split-off band at high energy with $\lambda = 600$ nm. The error bars correspond to the maximal observed scatter of $B_{\rm dp}$ around the corresponding mean values.

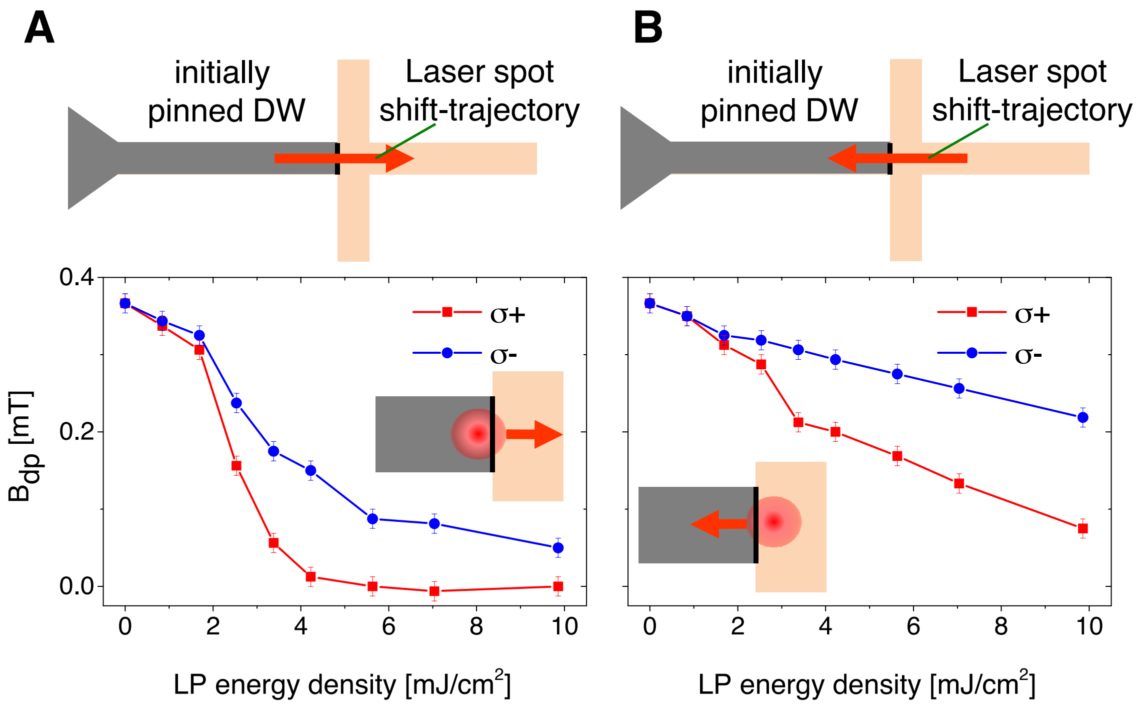

Supplementary Fig. 6: Sweep direction dependent domain wall depinning.

Depinning fields $B_{\mathrm{dp}}(\sigma^+)$ and $B_{\mathrm{dp}}(\sigma^-)$ versus LP energy density, (A), in case that the focused LP spot approaches from the narrow bar, and (B), when the LP spot approaches from the wider cross area. The error bars correspond to the maximal observed scatter of B_{dp} around the corresponding mean values.

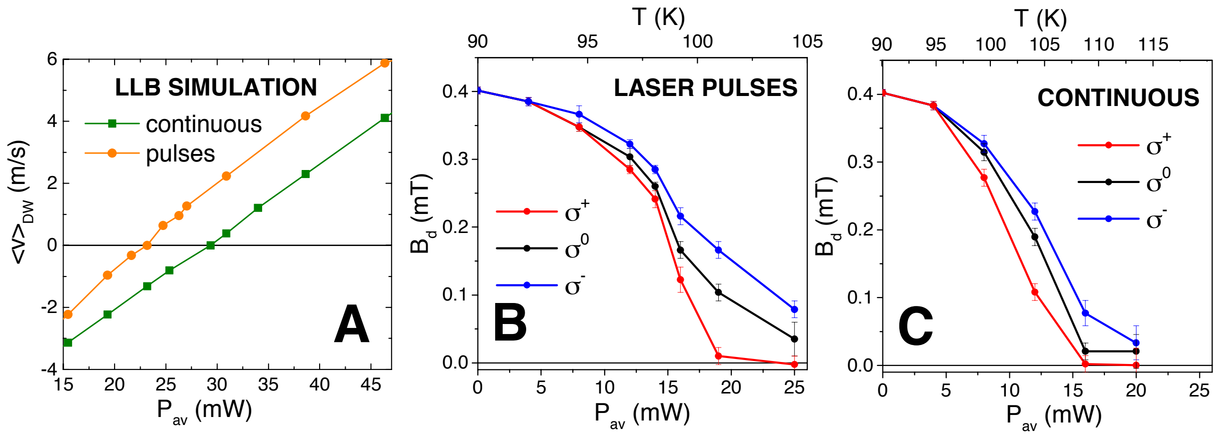

Supplementary Fig. 7: Ultrashort pulse excitation vs. continuous cw laser excitation

(A) Averaged DW velocities vs. P_{av} at the position of maximal restoring field for DW propagation driven by oSTT with ultrashort LP excitation (orange) and cw-excition (green). The averaged velocity is deduced from simulations of DW propagation based on the LLB approach. Depinning field B_{dp} vs. P_{av} for circularly (σ^+), (σ^-) and linearly polarized (σ^0) laser light in case of LP- (B) and cw-(C) excitation. We have assigned an effective sample temperature deduced from the comparison between temperature dependent measurements of B_{dp} without laser irradiation and measurements of $B_{dp}(\sigma^0)$ vs. P_{av} laser power and at fixed base temperature. The error bars correspond to the maximal observed scatter of B_{dp} around the corresponding mean values.

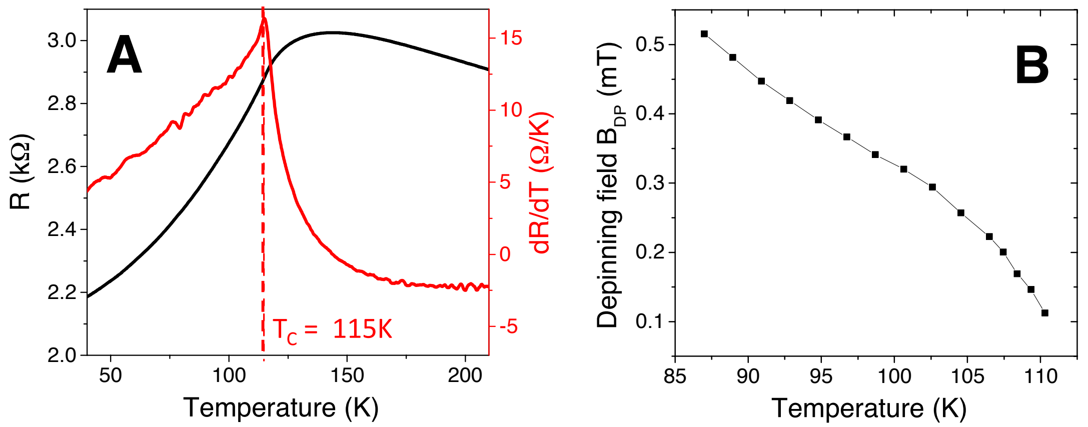

Supplementary Fig. 8: Magnetic properties of the GaMnAsP film.

(A) Temperature dependence of the resistance of a GaMnAsP bar and dR/dT identifying a Curie temperature of $Tc = 115$ K. (B) Depinning field B_{dp} without laser irradiation as a function of temperature.

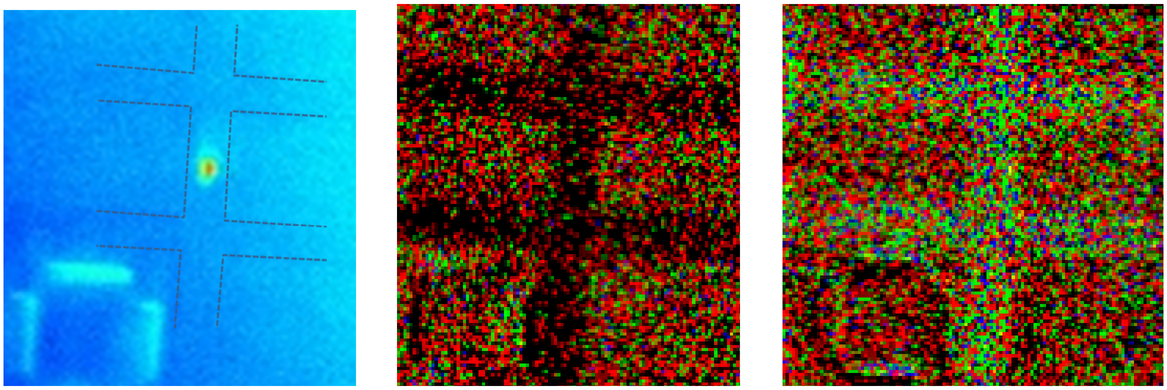

Supplementary Fig. 9: Experimental setup for MCD measurements.

Cross bar device for 4-point measurements: Left: Laser spot focused to the center of the bar between the two cross-contacts; Middle, (Right): MOKE micrographs at positive, (negative) magnetisation orientations.

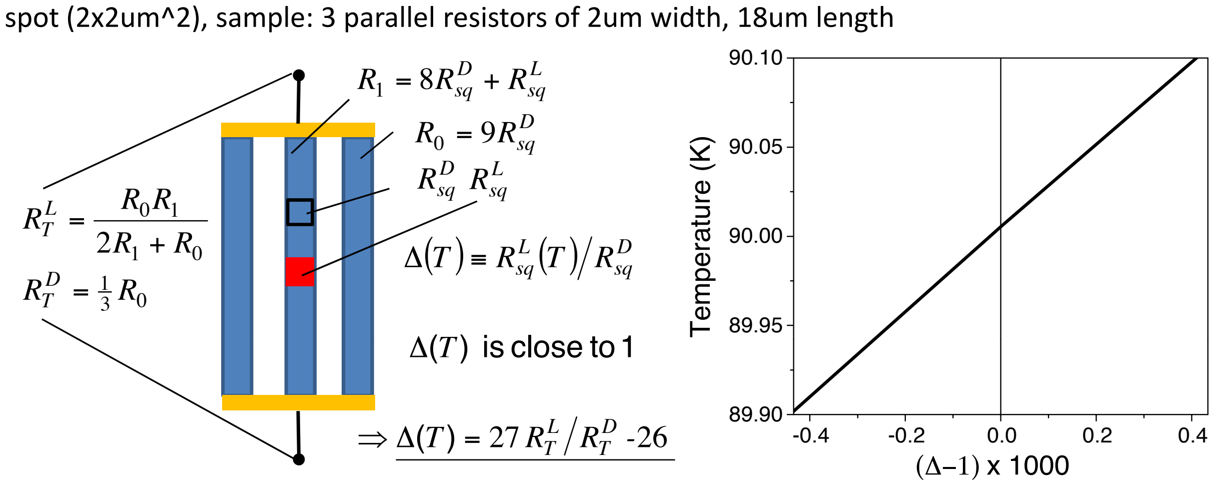

Supplementary Fig. 10: Model for MCD generated temperature variations.

Left: Simple resistor network model; Right: Resistance variations as a function of the sample temperature.

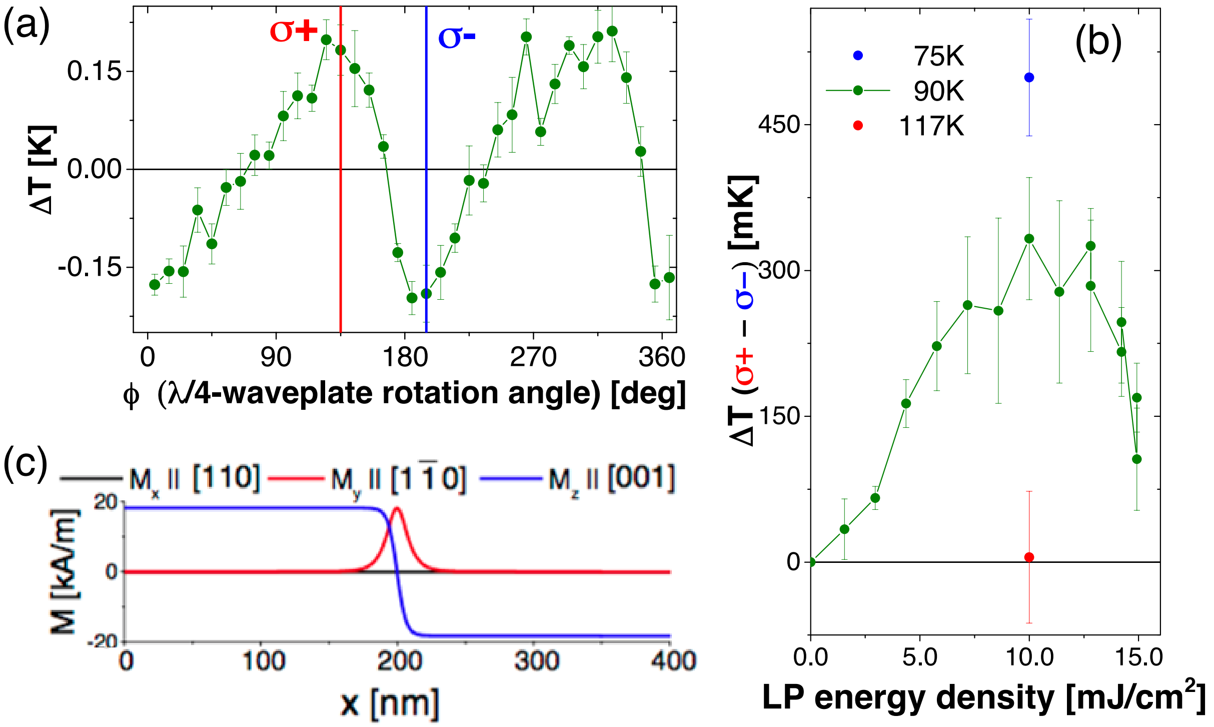

Supplementary Fig. 10: MCD induced temperature variations.

(a): Temperature variation measured at $T = 90$ K substrate temperature and for a laser power of 10 mJ/cm^2 as a function of LP polarisation. The polarisation is varied by a λ/4-waveplate. A constant offset is subtracted from all data points. Before taking a data point, we always realigned the focused laser spot to the bar center. The discrepancy between the measured angular dependence of the temperature variation from the expected $\cos(2\phi)$ behaviour is due to unintentional polarisation effects in some of the optical components in our setup. σ^+ and σ^- polarised LPs correspond to ϕ = 135 deg and ϕ = 195 deg, respectively. As expected for MCD related heating, we find largest temperature variations for σ^+ (red line) and for σ^- (blue line) polarisations. (b) MCD induced differences in temperature variation between σ^+ and σ^- as a function of LP energy density for 3 different substrate temperatures, 75 K (blue), 90 K (green), and 117 K (red). Note that the MCD induced heating disappears above the Curie temperature T_C =115 K. The error bars correspond to the standard deviation of the detected resistance variation. (c) Static DW profiles obtained by micromagnetic simulation with 0.5 nm (2 nm) discretisation length along (perp. to) a 4 μm wide bar. The simulation is based on the experimentally obtained values at T =90 K for out-of-plane uniaxial anisotropy $K_\perp$ =1500 Jm^{-3}, saturation magnetization M_S =18.2 kA/m and we use A =50 fJ/m for the exchange stiffness parameter.

Supplementary References

1. A. P. Malozemoff, J. C. Slonczewski: *Magnetic Domain Walls in Bubble Materials*, Academic Press, New York (1979).

2. J. Wunderlich: *Extraordinary Hall Effect in Multi-layered Magnetic Films: Application to the Study of Magnetization Reversal Dynamics*, ISBN-13: 978-3826591105, Shaker Verlag, Germany (2001).

3. C. Schieback, *et al.*, Temperature dependence of the current-induced domain wall motion from a modified Landau-Lifshitz-Bloch equation, *Phys. Rev. B.* **80**, 214403 (2009).

4. P. Němec, *et al.*, Experimental observation of the optical spin transfer torque, *Nat. Phys.* **8**, 411 - 415 (2012).

5. E. De Ranieri, *et al.*, Piezoelectric control of the mobility of a domain wall driven by adiabatic and non-adiabatic torques, *Nat. Mater.* **12**, 808 - 814 (2013).

6. A. J. Newell, W. Williams, D. J., Dunlop, A generalization of the demagnetizing tensor for nonuniform magnetization, J. Geophys. Res., **98** 9551-9555 (1993).

7. G. E. Pikus, A. N. Titkov, in *Optical Orientation*, F. Meier, B. P. Zakharchenya, Eds. (North-Holland, Amsterdam, 1984), p. 73.

8. V. Novák, *et al.*, Curie Point Singularity in the Temperature Derivative of Resistivity in (Ga,Mn)As, Phys. Rev. Lett. **101**, 077201 (2008).

9. S. Shihab, *et al.*, Steady-state thermal gradient induced by pulsed laser excitation in a ferromagnetic layer, J. Appl. Phys. **119**, 153904 (2016).
